# Long-Term Clinical Impact of Patients with Multi-Vessel Non-Obstructive Coronary Artery Disease

**DOI:** 10.3390/life13112119

**Published:** 2023-10-26

**Authors:** Jin Jung, Su-Nam Lee, Sung-Ho Her, Ki-Dong Yoo, Keon-Woong Moon, Donggyu Moon, Won-Young Jang

**Affiliations:** Department of Cardiology, St. Vincent’s Hospital, College of Medicine, The Catholic University of Korea, Seoul 16247, Republic of Korea; colaking@naver.com (J.J.); hhhsungho@naver.com (S.-H.H.); yookd@catholic.ac.kr (K.-D.Y.); cardiomoon@gmail.com (K.-W.M.); babaheesu@gmail.com (D.M.); raph83@naver.com (W.-Y.J.)

**Keywords:** non-obstructive coronary artery disease, multi-vessel, stroke, long-term clinical outcome, major cardiovascular and cerebrovascular event, myocardial infarction, cardiac death

## Abstract

Background: Non-obstructive coronary artery disease (CAD) is a disease commonly diagnosed in patients undergoing coronary angiography. However, little is known regarding the long-term clinical impact of multi-vessel non-obstructive CAD. Therefore, the object of this study was to investigate the long-term clinical impact of multi-vessel non-obstructive CAD. Method: A total of 2083 patients without revascularization history and obstructive CAD were enrolled between January 2010 and December 2015. They were classified into four groups according to number of vessels involved in non-obstructive CAD (25% ≤ luminal stenosis < 70%): zero, one, two, or three diseased vessels (DVs). We monitored the patients for 5 years. The primary outcome was major cardiovascular and cerebrovascular events (MACCEs), defined as a composite of cardiac death, stroke, and myocardial infarction (MI). Result: The occurrence of MACCEs increased as the number of non-obstructive DVs increased, and was especially high in patients with three DVs. After adjustment, patients with three DVs still showed significantly poorer clinical outcomes of MACCEs, stroke, and MI compared those with zero DVs. Conclusion: Multi-vessel non-obstructive CAD, especially in patients with non-obstructive three DVs, is strongly associated with poor long-term clinical outcomes. This finding suggests that more intensive treatment may be required in this subset of patients.

## 1. Introduction

In the past few decades, the prevalence of coronary artery disease (CAD) has been increasing continuously due to population growth and aging. Therefore, CAD remains the leading cause of morbidity and mortality worldwide, and the importance of the early diagnosis of CAD is increasing [1,2]. Consistent with this trend, coronary angiography (CAG) is actively used as a gold standard diagnostic tool. In addition, the use of coronary computerized tomography angiography (CCTA), instead of the use of the electrocardiogram test [3,4], allows the identification of many patients with mild to moderate stenosis.

Although non-obstructive CAD, including mild to moderate stenosis, is a relatively common finding in patients undergoing CAG and CCTA [5,6,7], non-obstructive CAD has been characterized as “insignificant CAD” in previous studies because of the low incidence of adverse outcomes [7,8]. Therefore, because most previous studies on CAD focused on patients with obstructive CAD, the risks associated with non-obstructive CAD were underestimated [9,10]. For this reason, management is well established for patients who have undergone percutaneous coronary intervention (PCI) or patients with obstructive CAD [3]. In particular, for statin treatment, different obvious target values are presented depending on the risk level, and for antiplatelet treatment, the combination, intensity, and duration are also obviously presented [11,12]. On the other hand, management for primary prevention in patients with non-obstructive CAD has not yet been established.

However, a prior study suggested that myocardial infarction (MI) occurred frequently in patients with non-obstructive lesions [13], and subsequent studies have supported an association between non-obstructive CAD and poor clinical outcomes [14,15]. Therefore, there is a growing awareness of the importance of non-obstructive CAD, and many studies are being conducted. However, the long-term clinical impact of multi-vessel non-obstructive CAD is still poorly understood. Therefore, the object of this study was to investigate the long-term clinical effect of multi-vessel non-obstructive CAD.

## 2. Materials and Methods

### 2.1. Study Design and Population

**Figure 1:**
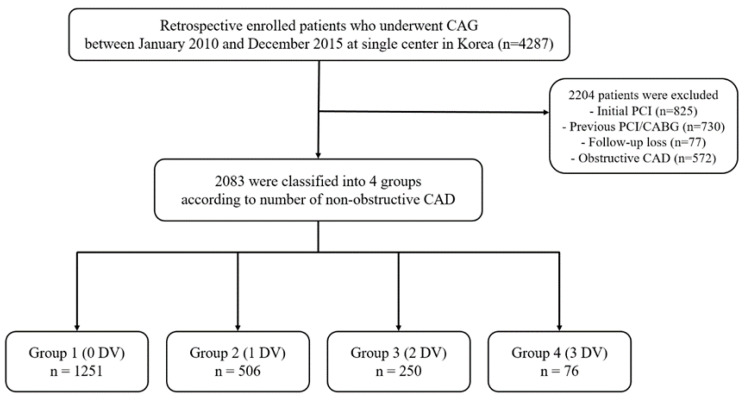
Study population flow chart. DV, diseased vessel; CAG, coronary angiography; CAD, coronary artery disease; PCI, percutaneous coronary intervention; CABG, coronary artery bypass grafting.

This was a non-randomized, retrospective, single-center study. We reviewed the medical records of 4287 patients who underwent CAG in St. Vincent Hospital, Suwon, Republic of Korea between January 2010 and December 2015. The angiographic findings, including degree of stenosis and extent of CAD, were evaluated visually by the attending interventional cardiologist. Referring to the standard definition used in a previous study and guidelines [14,16], obstructive CAD was defined as 50% or greater stenosis in the left main (LM) coronary artery or 70% or greater in other coronary arteries. Non-obstructive CAD was defined as CAD with mild (25–49%) to moderate (50–69%) stenosis, but LM CAD was defined as 20% or greater but less than 50% stenosis. Of the total patients, 2204 were excluded, including patients undergoing initial PCI, patients with previous PCI/coronary artery bypass graft history, and those with follow-up loss or obstructive CAD (Figure 1). The remaining 2083 patients were classified into 4 groups according to number of epicardial coronary arteries with non-obstructive CAD at the time of initial CAG: zero diseased vessels (DV) (0 DV), one diseased vessel (1 DV), two diseased vessels (2 DV), and three diseased vessels (3 DV). Patients with isolated non-obstructive LM disease were classified as 2 DV.

This study was approved by Institutional Review Board (IRB) of St. Vincent hospital (IRB VC20RISI0087) and complied with 1975 Declaration of Helsinki. The need for individual patient consent was waived.

### 2.2. Study Endpoint and Definition

The primary outcome of the study was major cardiovascular and cerebrovascular events (MACCEs), defined as a composite of cardiac death, stroke, and MI. The secondary outcomes were all-cause death, cardiac death, MI, and stroke. Because patients who initially underwent PCI were excluded from the study, MI referred to only spontaneous MI, defined as any troponin or creatine kinase-myocardial band increase above the upper limit of the normal range with ischemic signs or symptoms during the follow-up period after discharge, and periprocedural MI was excluded. Stroke was defined as neurological symptoms associated with radiologic evidence based on magnetic resonance imaging or computed tomography. The time-to-event duration was determined as that between study enrollment and the first event. Smoking was defined as having smoked cigarettes within 3 months of admission [17]. Chronic kidney disease (CKD) was defined as an estimated glomerular filtration rate <60 mL/min/1.73 m^2^, as calculated using the Modification of Renal Diet equation from baseline serum creatinine [18]. All clinical events were confirmed by source documentation collected at each hospital and centrally adjudicated by an independent group of clinicians unaware of the revascularization type.

### 2.3. Statistical Analyses

Continuous variables were presented as median and interquartile range or mean ± standard deviation and analyzed using Student’s t-test or Mann–Whitney test. Categorical variables were summarized as counts (percentages) and compared using the chi-square test or Fisher’s exact test, as appropriate. Event rates were calculated based on the Kaplan–Meier estimates in time-to-first event analysis and compared using the log-rank test. Univariable and multivariable Cox regression analyses were performed to analyze the clinical outcomes. The hazard ratio (HR) and 95% confidence interval (CI) were also calculated. Multivariable Cox regression models were adjusted for age, sex, hypertension (HTN), diabetes mellitus (DM), cerebrovascular accident (CVA), intravascular ultrasonography (IVUS), fractional flow reserve (FFR), aspirin, P2Y12 inhibitor, cilostazol, statin, vasodilator, left ventricular ejection fraction (LV EF), and creatinine.

All statistical analyses were conducted using Statistical Analysis Software (SAS, version 9.2, SAS Institute, Cary, NC, USA) and *p*-value < 0.05 was considered statistically significant.

## 3. Results

During the study period, 2083 patients with non-obstructive CAD or 0 DV were analyzed. Of those, 1251 (60%) were classified as 0 DV, and the remaining patients were divided into three groups according to number of non-obstructive DVs (1 DV: 506 [24% of total patients], 2 DV: 250 [12%], 3 DV: 76 [4.0%]).

### 3.1. Baseline Characteristics

Baseline characteristics are displayed in Table 1. Compared to patients with low burden of atherosclerotic disease (non-obstructive 0 DV and 1 DV), older age and prevalence of HTN and DM were higher in patients with multi-vessel non-obstructive CAD (age, 0 DV: 59.2 ± 11.9, 1 DV: 64.1 ± 10.7, 2 DV: 65.8 ± 11.1, 3 DV: 65.1 ± 12.9, *p* < 0.001; HTN, 0 DV: 561 [44.8%], 1 DV: 274 [54.2%], 2 DV: 162 [64.8%], 3 DV: 53 [69.7%], *p* < 0.001; DM, 0 DV: 231 [18.5%], 1 DV: 126 [24.9%], 2 DV: 81 [32.4%], 3 DV: 23 [30.3%], *p* < 0.001). Moreover, previous CVA was most frequent in patients with 3VD (Previous CVA, 0 DV: 83 [6.6%], 1 DV: 33 [6.5%], 2 DV: 26 [10.4%], 3 DV: 15 [19.7%], *p* < 0.001). The rates of aspirin, P2Y12 inhibitor, and statin medication use related to these underlying diseases showed the same tendency (aspirin, 0 DV: 385 [30.8%], 1 DV: 278 [54.9%], 2 DV: 177 [70.8%], 3 DV: 51 [67.1%], *p* < 0.001; P2Y12 inhibitor, 0 DV:46 [3.7%], 1 DV: 45 [8.9%], 2 DV: 29 [11.6%], 3 DV: 10 [13.2%], *p* < 0.001; Statin, 0 DV: 276 [22.1%], 1 DV: 184 [36.4%], 2 DV: 104 [41.6%], 3 DV: 31 [40.8%], *p* < 0.001). Laboratory and procedural characteristics showed no difference in groups except creatinine and FFR usage (creatinine, 0.9 ± 0.9, 1 DV: 1.4 ± 1.7, 2 DV: 1.0 ± 0.8, 3 DV: 1.5 ± 1.7, *p* = 0.046; FFR, 0 DV: 0 [0.0%], 1 DV: 7 [1.4%], 2 DV: 4 [1.6%], 3 DV: 0 [0.0%], *p* < 0.001).

### 3.2. Long-Term Clinical Outcomes

**Figure 2:**
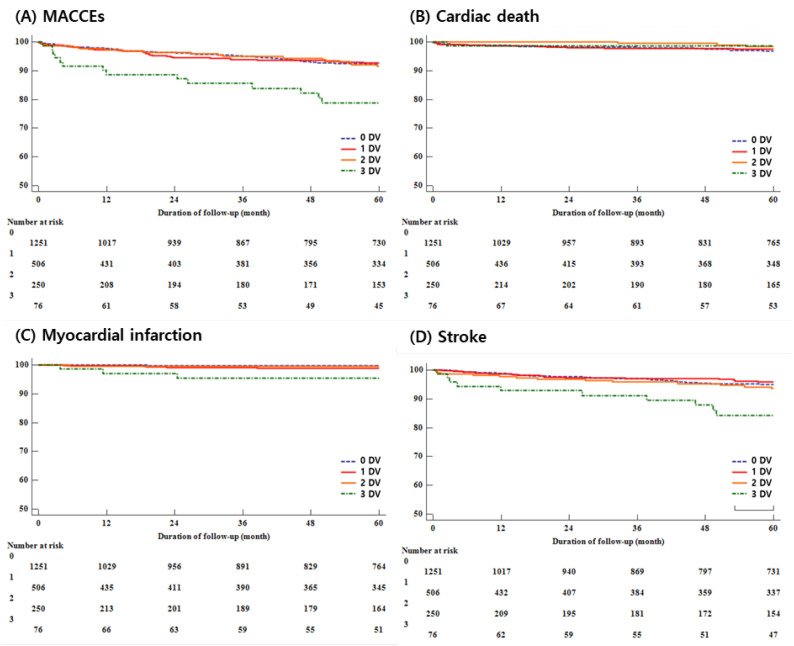
(**A**) Major cardiovascular and cerebrovascular events; (**B**) Cardiac death; (**C**) Myocardial infarction; (**D**) Stroke.

All-cause death and cardiac death did not show statistically significant differences in the groups (All-cause death, 0 DV: 118 [9.4%], 1 DV: 45 [8.9%], 2 DV: 28 [11.2%], 3 DV: 14 [18.4%], *p* = 0.680; Cardiac death, 0 DV: 31 [2.5%], 1 DV: 11 [2.2%], 2 DV: 3 [1.2%], 3 DV: 1 [1.3%], *p* = 0.597), but they were significantly different in patients with MACCEs, MI, or stroke (MACCEs, 0 DV: 78 [6.2%], 1 DV: 33 [6.5%], 2 DV: 17 [6.8%], 3 DV: 14 [18.4%], *p* < 0.001; MI, 0 DV: 3 [0.2%], 1 DV: 5 [1.0%], 2 DV: 1 [0.4%], 3 DV: 3 [4.0%], *p* < 0.001; Stroke, 0 DV: 43 [3.9%], 1 DV: 18 [3.6%], 2 DV: 13 [5.2%], 3 DV: 10 [13.2%], *p* = 0.001). The occurrence of MACCEs increased as the number of non-obstructive DVs increased and, in particular, with a nearly 3 times higher occurrence rate in the 3 DVs compared to the 2 DVs group. In addition, the occurrence of MACCEs was mainly due to stroke rather than cardiac events (Table 2) (Figure 2).

In multivariable analysis, patients with non-obstructive 3 DVs showed significantly worse long-term clinical outcomes than those with 0 DVs in terms of MACCEs, stroke, and MI (MACCEs: hazard ratio [HR] 2.09, 95% confidence interval [CI] 1.15–3.79, *p* = 0.016; Stroke: HR 2.04, 95% CI 1.01–4.16, *p* = 0.049, MI: HR 15.17, 95% CI 2.37–97.25, *p* = 0.004) (Table 3).

## 4. Discussion

The main findings of the study were as follows: (1) Non-obstructive CAD was associated with adverse long-term clinical outcomes, especially in patients with non-obstructive three DVs; as the number of non-obstructive DVs increased, the frequency of 5-year MACCE increased; (2) Compared to patients without non-obstructive DV, patients with three DVs showed twice the risk of MACCE and stroke and 15 times the risk of MI; (3) The occurrence of MACCE was primarily due to stroke and not cardiac events.

The population in the present study had no previous history of revascularization and were diagnosed with non-obstructive CAD after initial CAG and did not undergo PCI. These patients are often encountered in clinical practice, while clinical relevance may be underestimated as they are considered low risk for adverse events and clear guidelines for the management of these patients have not been established. Currently, FFR is used in addition to angiography for therapeutic decisions in these patients. However, in many cardiac laboratories, it is still common to base a clinical decision on coronary angiography findings alone due to equipment availability, financial considerations, and insurance issues. In that respect, our study was similar to real-world clinical practice.

Our results show that non-obstructive CAD is associated with poor clinical outcomes, consistent with several previous studies showing such a negative impact [14,15,19,20] and with prior studies indicating that a majority of plaque ruptures and MIs are related to non-obstructive rather than obstructive lesions [9,13,21]. In addition, as the number of obstructive vessels increases, the clinical outcomes worsen, especially in patients with obstructive three DVs [6,9,14]. Through this study, the same trend was confirmed in non-obstructive CAD. These results are also consistent with the results of previous studies that evaluated CAD with CCTA [22,23]. In a previous study [22] of patients with no history of PCI, patients with a small number of segments with disease did not differ from patients without non-obstructive CAD when comparing survival free from cardiovascular death or MI. However, among patients with non-obstructive CAD, those with extensive segments with disease experienced a higher rate of cardiovascular death or myocardial infarction, comparable with those who have a small number of segments with disease and showed similar clinical outcomes to patients with obstructive CAD. In another study [23] of a similar patient group, patients with non-obstructive CAD had a 6% higher risk of death for each additional segment with non-obstructive plaque. These studies have shown the importance of not only the presence of non-obstructive CAD but also its extent. This conclusion is in line with our study, where the number of adverse events increased as the number of non-obstructive DVs increased.

Patients with multi-vessel non-obstructive CAD were older, had a higher female proportion, and had a higher co-morbid incidence including DM, HTN, and CVA. These baseline characteristic differences were more evident between one DV and two DVs than between two DVs and three DVs, and have shown the same trend in a previous major study [14]. In particular, the female ratio of two DVs was similar to three DVs, but higher than one DV. Gender may have influenced these differences because female patients show higher HTN, DM incidence than their male counterparts [24]. Age was known to be a strong predictor of adverse events in non-obstructive CAD patients, and so was the DM [25]. In the presence of DM, both non-obstructive CAD and obstructive CAD patients had poorer clinical outcomes than patients without DM, and diabetic patients with non-obstructive CAD had the worse outcome, which was comparable to patients with obstructive CAD alone [26]. Hypertension was well known as a cardiovascular risk factor and was associated with the extent of non-obstructive CAD and triple vessel disease [27]. Prior CVA was also independently associated with a higher risk of MACCEs [28]. Therefore, these factors may have affected the present study results. However, after adjusting for these factors, non-obstructive three DVs remained an independent predictor of MACCEs, unlike one DV and two DVs. These results were the same as those of Maddox et al. [14], who presented short-term (1-year) outcomes of similar patients using similar definitions of non-obstructive CAD. Our study confirmed that this trend was maintained in the long term (5 years).

Contrary to age, gender, and co-morbidity, there were no differences between groups in terms of laboratory characteristics. In particular, CPR, a representative inflammation marker, was well known as a marker that can predict cardiovascular events in previous studies [29,30], but no difference was found in this study. Previous studies targeted a selective population such as patients with typical chest pain and evidence of ischemia [29] or patients with a high risk of atherosclerotic disease [30]. On the other hand, this study was a retrospective study that excluded patients with a history of previous revascularization and consisted of a less selective population. Therefore, the study population may have had an influence. Additionally, the association between CRP and cardiovascular events demonstrated statistical significance, but the association with stroke did not [31]. In our study, MACCEs occurred more frequently in stroke than in cardiac events, so this may also have had an impact.

There was a statistically significant difference in coronary involvement in all groups. In particular, the location of the lesion is important in patients with non-obstructive CAD, and it is well known that proximal coronary involvement was associated with increased adverse event risk in patients with non-obstructive CAD [32]. Our study results also showed that proximal left anterior descending (LAD) involvement increased as DV increased and showed poor outcomes.

Even in non-obstructive CAD, multi-vessel non-obstructive CAD, reflecting a greater atherosclerotic burden, affected the poor clinical impact, suggesting that not only the degree of stenosis, but also the total atherosclerotic burden, are important factors in prognosis. Previous studies have reported that the burden of atherosclerotic disease is a strong predictor of prognosis [33,34,35]. A study using CCTA, another diagnostic modality, also showed poor clinical outcomes, as calcified plaque burden and non-calcified plaque burden increased independently of CAD severity [36]. Maddox et al. [14], mentioned above, showed that, as CAD extent increased, the clinical outcome, defined as a composite of MI and mortality, worsened. In obstructive CAD, HR progressively increased as the number of involved vessels increased. The same trend was seen in non-obstructive CAD, but it was statistically significant only in three DVs. A similar result was also demonstrated in a study evaluating CAD extent with CCTA [37]. Mortensen et al. [15] demonstrated that patients with comparable total atherosclerotic burden had a similar risk for cardiovascular events regardless of non-obstructive or obstructive CAD. This indicates that the main predictor of clinical outcome is not the degree of stenosis, but the total atherosclerotic burden, which would have had a significant impact on the derivation of our findings.

Endothelial dysfunction may also have influenced the result that patients with multi-vessel non-obstructive CAD showed a poorer clinical outcome. The vascular endothelium plays an important role in vascular tone and flow, as well as permeability and thrombosis homeostasis. Therefore, endothelial dysfunction causes a decline in these functions, resulting in a decrease in anti-atherosclerotic and anti-thrombotic properties [38]. In other words, endothelial dysfunction is a marker of atherosclerosis and itself contributes to the progression of atherosclerosis [39]. A large cohort study with 5 years of follow-up has already shown that endothelial dysfunction was a major predictor of MACCE, defined identically to our study [40], and, as in our study, the presence of endothelial dysfunction increased cardiac events even in patients with non-obstructive CAD [41]. Such endothelial dysfunction worsened as the number of DVs increased [41,42], which is consistent with the present study, showing that patients with multi-vessel non-obstructive CAD had worse clinical outcomes. The presence of endothelial dysfunction in patients with non-obstructive CAD predicts adverse cardiovascular outcomes, but careful interpretation is necessary because endothelial dysfunction is not always present in non-obstructive CAD.

Gender difference may also have contributed to poor prognosis with multi-vessel non-obstructive CAD. Contrary to obstructive CAD, the prevalence of non-obstructive CAD is higher in women, but, paradoxically, women show higher rates of myocardial ischemia and mortality than men [43,44]. Our study also showed a higher female ratio, especially in multi-vessel CAD, which is the same result as the previous study [24]. The reason why females have a worse outcome is not yet fully explained, but it is thought that the higher frequency of inflammatory disease and microvascular dysfunction, adverse pregnancy outcome, and hormonal and menopause effects may have had an effect [44,45].

Many cardiologists focus only on significant CAD in patients who have undergone CAG. However, given that atherosclerosis is a systemic process and the prevalence of poly-vascular atherosclerosis is common [46,47], the potential risk of other atherosclerosis should be considered. In particular, CAD often coexists with stroke because they share risk factors and pathogenesis [48,49] and the extent of CAD is associated with an incremental risk of stroke. In our study, the overall CVA prevalence among patients with non-obstructive CAD including one DV, two DVs, and three DVs was 8.9% (74/832), which is much higher than the prevalence of 3.72% for general Koreans of a similar age group [50]. This figure is similarly confirmed in the CVA prevalence (9.8%) in another study targeting Koreans with non-obstructive CAD [51]. In the present study, the occurrence of stroke but not a cardiac event contributed most to the occurrence of MACCE. These results have important implications for East Asian countries such as Korea, because stroke has a higher mortality than CAD in East Asia, opposed to findings in Western and other Asian countries [49].

Our results suggest that the total atherosclerotic burden is a more important factor in long-term prognosis than the dichotomous definition of CAD as obstructive or non-obstructive. That is, even non-obstructive CAD is associated with a worse prognosis if it is multi-vessel, especially non-obstructive three DVs. However, there are no clear guidelines for the management of patients with non-obstructive CAD, and there is lack of awareness that intensive treatment should be provided. Recently, management for the primary prevention of non-obstructive CAD patients has been studied [23,52]. In particular, a large-scale study [23] showed that aspirin had no benefit in patients with non-obstructive CAD, whereas statin was associated with a significant reduction in mortality for individuals with non-obstructive CAD. However, further research is still needed on the statin intensity or target level. In addition, non-obstructive CAD patients also have different effects across subgroups [51], requiring research on which patient groups are effective. Considering the results of our study, clinicians should consider intensive medical care when treating patients with multi-vessel non-obstructive CAD.

## 5. Limitations

The present study had several limitations. First, as a retrospective and observational study, selection bias could have contributed to the results. Second, since the degree of CAD stenosis and distribution were evaluated subjectively, there was a possibility of misclassification. However, this would have been offset by the performance in a single center. Third, plaque location such as proximal or distal and characteristics could affect MACCEs, but only the proximal LAD was analyzed, and the proximal left circumflex artery and right coronary artery involvement were not analyzed. Fourth, in patients with non-obstructive CAD, coronary artery spasm may have been one of the causes leading to sudden cardiac death and myocardial infarction, which may have influenced the results showing poor prognosis. However, coronary artery spasm was not investigated. Finally, despite a follow-up of 5 years, the occurrence of cardiac events was relatively small, so there was a limit to the interpretation. Therefore, data for a longer period are needed.

## 6. Conclusions

Multi-vessel non-obstructive CAD, reflecting higher atherosclerotic burden, is associated with poor long-term (5 years) clinical outcomes, mainly stroke. These findings suggest that more intensive treatment may be required in this subset of patients, and the risk of stroke, as well as of cardiovascular events, should be considered. Further large-scale prospective studies are needed to determine proper management to prevent future events in these patients.

## Figures and Tables

**Table 1:** Baseline demographic and clinical characteristics.

	0 DV n = 1251	1 DV n = 506	2 DV n = 250	3 DV n = 76	*p*-Value
Age (years)	59.2 ± 11.9	64.1 ± 10.7	65.8 ± 11.1	65.1 ± 12.9	<0.001
Male	629 (50.3)	241 (47.6)	101 (40.4)	32 (42.1)	0.024
BMI	24.4 ± 3.6	24.6 ± 3.4	24.2 ± 3.1	24.3 ± 4.1	0.544
Smoking	353 (28.2)	158 (31.2)	78 (31.2)	24 (31.6)	0.529
HTN	561 (44.8)	274 (54.2)	162 (64.8)	53 (69.7)	<0.001
DM	231 (18.5)	126 (24.9)	81 (32.4)	23 (30.3)	<0.001
Dyslipidemia	229 (18.3)	109 (21.5)	57 (22.8)	17 (22.4)	0.216
CKD	23 (1.8)	14 (2.8)	7 (2.8)	2 (2.6)	0.574
Previous CVA	83 (6.6)	33 (6.5)	26 (10.4)	15 (19.7)	<0.001
LV EF (%)	59.3 ± 10.6	60.9 ± 8.9	61.6 ± 7.7	58.0 ± 11.8	0.013
Aspirin	385 (30.8)	278 (54.9)	177 (70.8)	51 (67.1)	<0.001
P2Y12 inhibitor	46 (3.7)	45 (8.9)	29 (11.6)	10 (13.2)	<0.001
Cilostazol	19 (1.5)	13 (2.6)	14 (5.6)	3 (4.0)	0.001
Statin	276 (22.1)	184 (36.4)	104 (41.6)	31 (40.8)	<0.001
Beta blocker	185 (14.8)	87 (17.2)	42 (16.8)	17 (22.4)	0.229
ACEi/ARB	242 (19.3)	105 (20.8)	67 (26.8)	15 (19.7)	0.068
Vasodilator	333 (26.6)	201 (39.7)	111 (44.4)	38 (50.0)	<0.001
IVUS	1 (0.1)	4 (0.8)	2 (0.8)	1 (1.3)	0.045
FFR	0 (0.0)	7 (1.4)	4 (1.6)	0 (0.0)	<0.001
HbA1c (%)	6.4 ± 1.2	6.6 ± 1.3	7.1 ± 1.7	6.3 ± 1.0	0.058
Proximal LAD	0 (0.0)	83 (16.4)	50 (20.0)	28 (36.8)	<0.001
LAD (lesion)	0 (0.0)	260 (51.4)	155 (62.0)	52 (68.4)	<0.001
Mild stenosis (lesion)	0 (0.0)	164 (32.4)	86 (34.4)	27 (35.5)	
Moderate stenosis (lesion)	0 (0.0)	96 (19.0)	69 (27.6)	25 (32.9)	
LCx (lesion)	0 (0.0)	65 (12.9)	109 (43.6)	67 (88.2)	<0.001
Mild stenosis (lesion)	0 (0.0)	42 (8.3)	56 (22.4)	30 (39.5)	
Moderate stenosis (lesion)	0 (0.0)	23 (4.6)	53 (21.2)	37 (48.7)	
RCA (lesion)	0 (0.0)	114 (22.5)	166 (66.4)	76 (100.0)	<0.001
Mild stenosis (lesion)	0 (0.0)	75 (14.8)	116 (46.4)	39 (51.3)	
Moderate stenosis (lesion)	0 (0.0)	39 (7.7)	50 (20.0)	37 (48.7)	
Creatinine (mg/dL)	0.9 ± 0.9	1.4 ± 1.7	1.0 ± 0.8	1.5 ± 1.7	0.046
Hemoglobin (g/dL)	13.6 ± 1.8	13.6 ± 1.9	13.6 ± 1.8	13.1 ± 2.2	0.738
Hematocrit (%)	39.6 ± 5.0	39.7 ± 4.6	39.8 ± 5.0	37.9 ± 5.8	0.876
Platelet (×10^9^/L)	238.9 ± 68.2	238.7 ± 63.5	236.3 ± 71.6	232.6 ± 76.5	0.853
White blood cell (×10^9^/L)	7.9 ± 10.6	7.8 ± 6.2	8.3 ± 4.3	7.3 ± 2.6	0.939
Total cholesterol (mg/dL)	181.1 ± 43.1	183.5 ± 41.8	186.9 ± 53.3	169.4 ± 38.0	0.649
Triglyceride (mg/dL)	129.7 ± 113.4	131.0 ± 81.0	142.7 ± 105.2	105.9 ± 59.2	0.485
HDL cholesterol (mg/dL)	43.2 ± 11.6	43.0 ± 11.8	40.8 ± 11.7	43.7 ± 12.1	0.346
LDL cholesterol (mg/dL)	107.8 ± 33.5	109.5 ± 35.6	106.5 ± 41.6	102.1 ± 29.6	0.240
C-Reactive protein (mg/dL)	1.1 ± 3.1	1.0 ± 5.1	1.8 ± 3.5	0.5 ± 0.6	0.116
Albumin	4.3 ± 1.2	4.4 ± 4.7	4.2 ± 0.6	4.2 ± 0.3	0.114

Data are expressed as mean ± standard deviation or number (%); DV, diseased vessel; BMI, body mass index; HTN, hypertension; DM, diabetic mellitus; CKD, chronic kidney disease; CVA, cerebrovascular accident; LV EF, left ventricular ejection fraction; ACEi, angiotensin-converting–enzyme inhibitor; ARB, angiotensin II receptor blocker; IVUS, intravascular ultrasonography; FFR, fractional flow reserve; HbA1c, glycated hemoglobin; LAD, left anterior descending artery; LCx, left circumflex artery; RCA, right coronary artery.

**Table 2:** Five-year clinical outcomes.

	0 DV n = 1251	1 DV n = 506	2 DV n = 250	3 DV n = 76	*p*-Value
MACCEs	78 (6.2)	33 (6.5)	17 (6.8)	14 (18.4)	<0.001
All-cause death	118 (9.4)	45 (8.9)	28 (11.2)	9 (11.8)	0.680
Cardiac death	31 (2.5)	11 (2.2)	3 (1.2)	1 (1.3)	0.597
Myocardial infarction	3 (0.2)	5 (1.0)	1 (0.4)	3 (4.0)	<0.001
Stroke	49 (3.9)	18 (3.6)	13 (5.2)	10 (13.2)	0.001

Data are shown as n (%); DV, diseased vessel; MACCEs, major adverse cardiovascular and cerebrovascular events.

**Table 3:** Five-year hazard ratio of MACCEs, all-cause death, cardiac death, myocardial infarction, and stroke.

	Univariate HR (95% CI)	*p*-Value	Multivariate HR ^a^ (95% CI)	*p*-Value
MACCEs				
0 DVs (reference)	1.00	-	1.00	-
1 DV	0.98 (0.65–1.48)	0.928	0.83 (0.54–1.27)	0.386
2 DVs	1.05 (0.62–1.78)	0.854	0.70 (0.40–1.23)	0.212
3 DVs	2.97 (1.68–5.24)	<0.001	2.09 (1.15–3.79)	0.016
All-cause death				
0 DVs (reference)	1.00	-	1.00	-
1 DV	0.87 (0.62–1.12)	0.434	0.77 (0.54–1.10)	0.148
2 DVs	1.11 (0.74–1.68)	0.612	0.85 (0.55–1.32)	0.466
3 DVs	1.13 (0.57–2.22)	0.734	0.77 (0.39–1.55)	0.469
Cardiac death				
0 DVs (reference)	1.00	-	1.00	-
1 DV	0.83 (0.42–1.65)	0.595	0.80 (0.39–1.65)	0.548
2 DVs	0.46 (0.14–1.51)	0.201	0.37 (1.11–1.29)	0.118
3 DVs	0.49 (0.07–3.60)	0.485	0.45 (0.06–3.35)	0.433
Myocardial infarction				
0 DVs (reference)	1.00	-	1.00	-
1 DV	3.92 (0.94–16.42)	0.061	3.31 (0.73–14.99)	0.120
2 DVs	1.60 (0.17–15.40)	0.683	1.44 (0.13–15.77)	0.765
3 DVs	15.65 (3.16–77.56)	<0.001	15.17 (2.37–97.25)	0.004
Stroke				
0 DVs (reference)	1.00	-	1.00	-
1 DV	0.85 (0.49–1.45)	0.540	0.67 (0.38–1.16)	0.152
2 DVs	1.27 (0.69–2.34)	0.441	0.74 (0.39–1.42)	0.362
3 DVs	3.30 (1.67–6.52)	<0.001	2.04 (1.01–4.16)	0.049

^a^ adjusted by age, sex, hypertension, diabetic mellitus, cerebrovascular accident, intravascular ultrasonography, fractional flow reserve, aspirin, P2Y12 inhibitor, cilostazol, statin, vasodilator, left ventricular ejection fraction, and creatinine. HR, hazard ratio; CI, confidence interval; DV, diseased vessel; MACCEs, major adverse cardiovascular and cerebrovascular events.

## Data Availability

The data presented in this study are available on request from the corresponding author.
